# Supplementary material for: Two New Nonaborates with {B9} Cluster Open-Frameworks and Short Cutoff Edges
Source: Molecules. 2022 Aug 18;27(16):5279. doi: 10.3390/molecules27165279 (PMC9416470; doi:10.3390/molecules27165279)
Supplement: Supplementary file 1 [file molecules-27-05279-s001.zip › molecules-1860973-supplementary.pdf]

Supplementary Materials

Two New Nonaborates with $\{B_9\}$ Cluster Open-Frameworks and Short Cutoff Edges

Chong-An Chen and Guo-Yu Yang *

MOE Key Laboratory of Cluster Science, School of Chemistry and Chemical Engineering, Beijing Institute of Technology, Beijing 100081, China; cca@bit.edu.cn

* Correspondence: ygy@bit.edu.cn or ygy@fjirsm.ac.cn

Table S1. Hydrogen bond distances (Å) and angles (°) for the compound **2**.

Figure S1. Porous layered structure (a) and the seven intercommunicated channels (b) in $[H_3O]K_{3.52}Na_{3.48}\{Al_2[B_7O_{13}(OH)][B_5O_{10}][B_3O_5]\}[CO_3]$.

Figure S2. (a) Metal positions in the framework of **1**; (b)Na_4BaO_{28} pentamers in **1**.

Figure S3. (a) Metal positions in framework **2**; (b) Metal-O monolayer in **2**; (c) Metal-oxygen porous layer in **2**.

Figure S4. PXRD of **1** (a) and **2** (b).

Figure S5. IR spectra of **1** and **2**.

Figure S6. TG curves of **1** and **2**.

Table S1. Hydrogen bond distances (Å) and angles (°) for the compound **2**.

D–H···A	d(D–H)	d(H···A)	d(D···A)	∠(DHA) (°)
O(7)–H(7)···O(8)#1	0.81	2.38	2.9314	126.0
O(7)–H(7)···O(9)#1	0.81	2.36	3.0801	149.6

Symmetric codes: #1: x,−y,−1/2 + z.

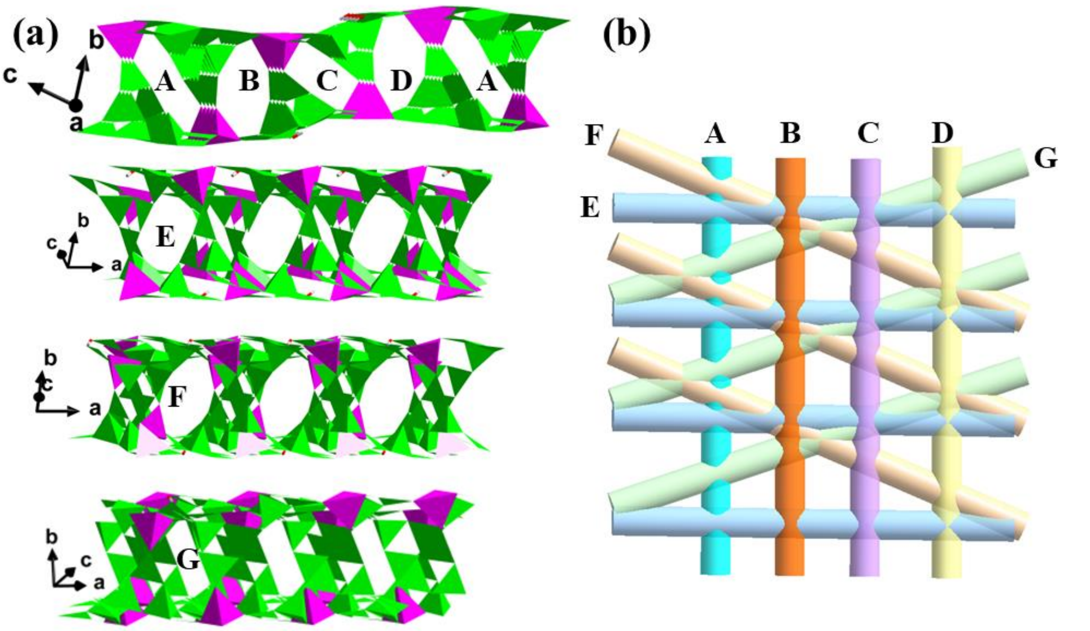

Figure S1. Porous layered structure (**a**) and the seven intercommunicated channels (**b**) in $[H_3O]K_{3.52}Na_{3.48}\{Al_2[B_7O_{13}(OH)][B_5O_{10}][B_3O_5]\}[CO_3]$.

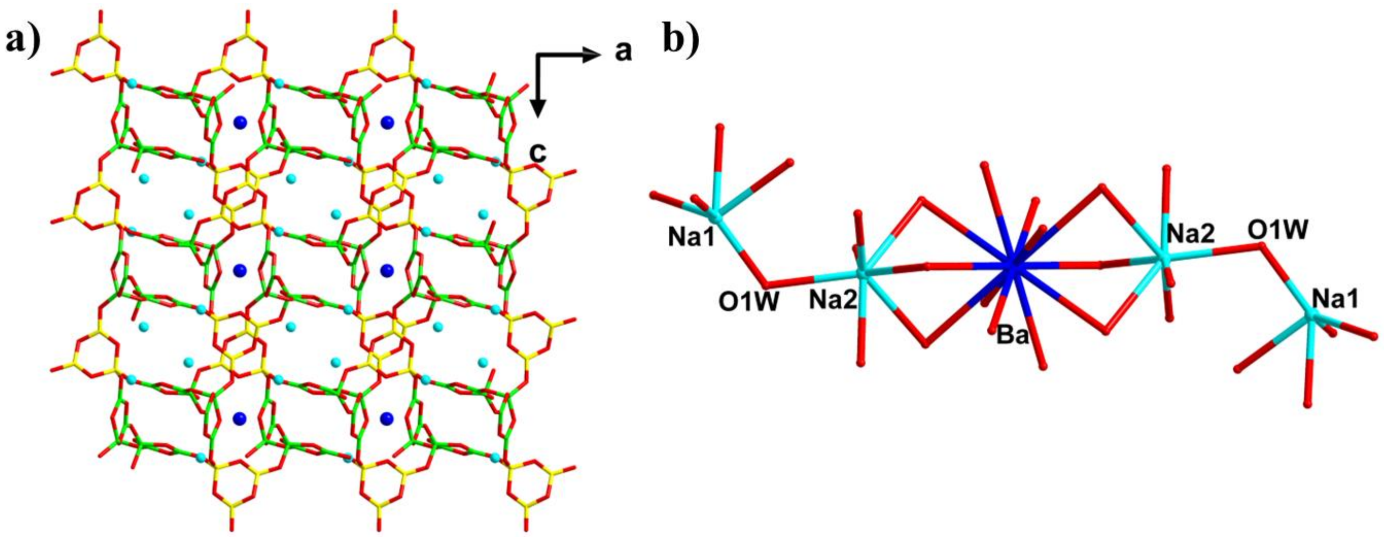

Figure S2. (**a**) Metal positions in the framework of **1**; (**b**) Na_4BaO_{28} pentamers in **1**.

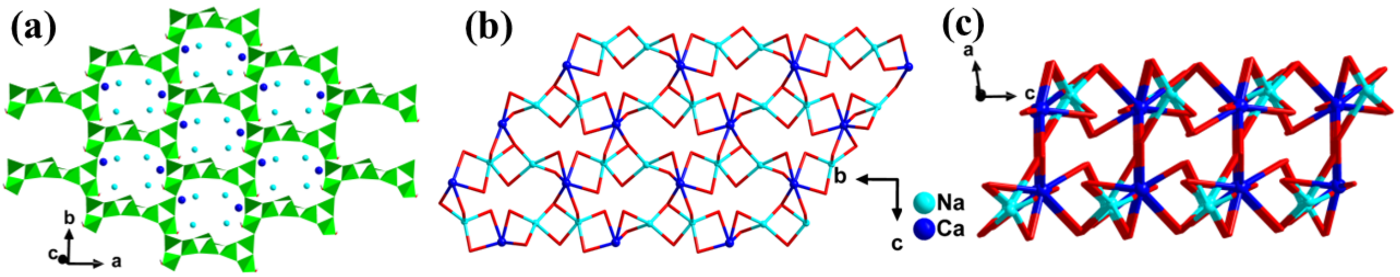

Figure S3. (**a**) Metal positions in the framework of **2**; (**b**) Metal-oxygen monolayer in **2**; (**c**) Metal-oxygen porous layer in **2**.

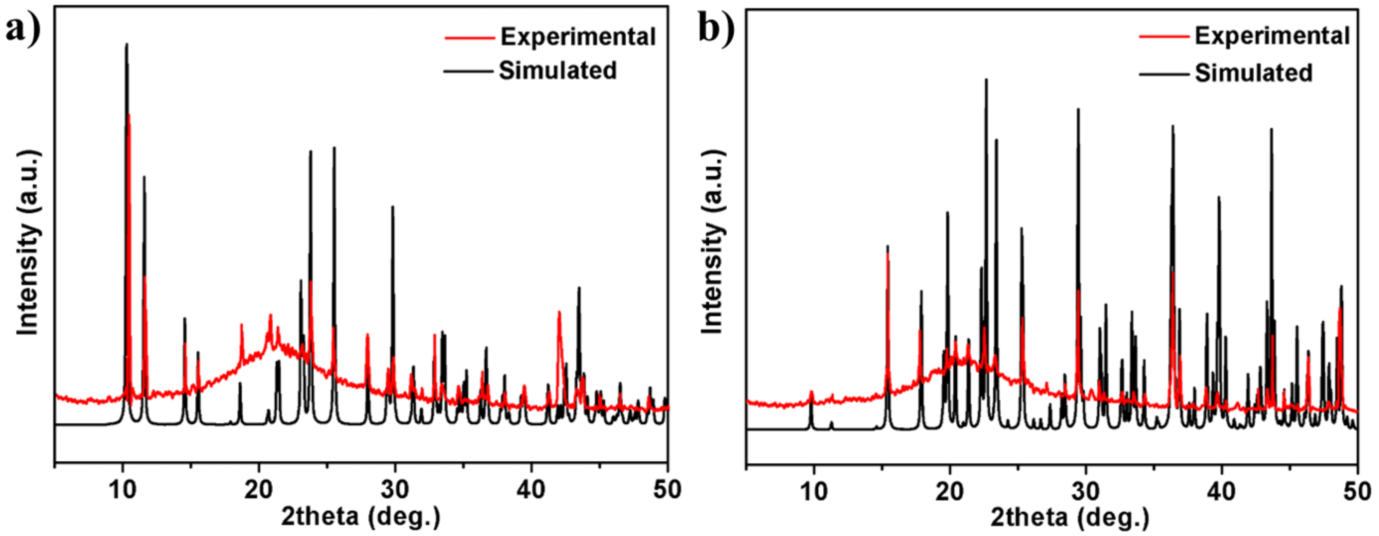

Figure S4. PXRD of **1** (**a**) and **2** (**b**).

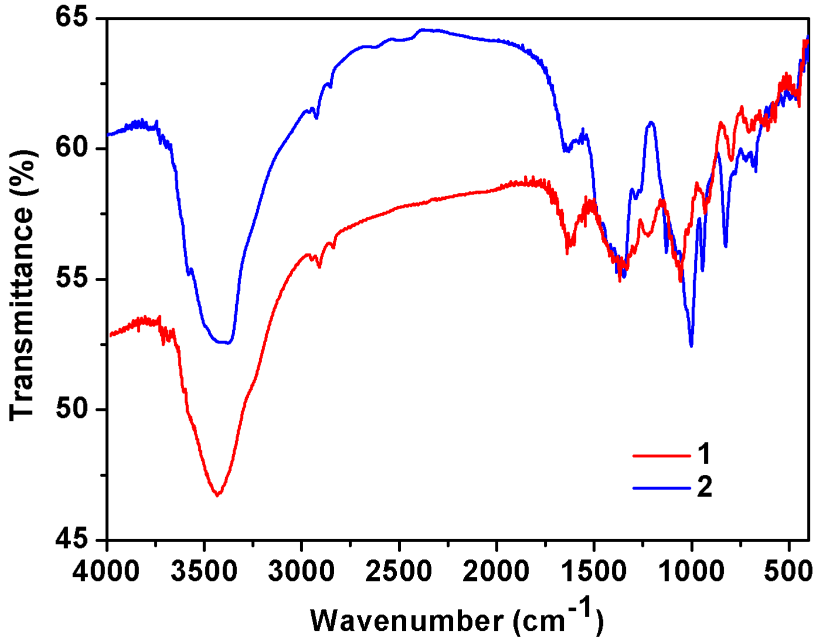

Figure S5. IR spectra of **1** and **2**.

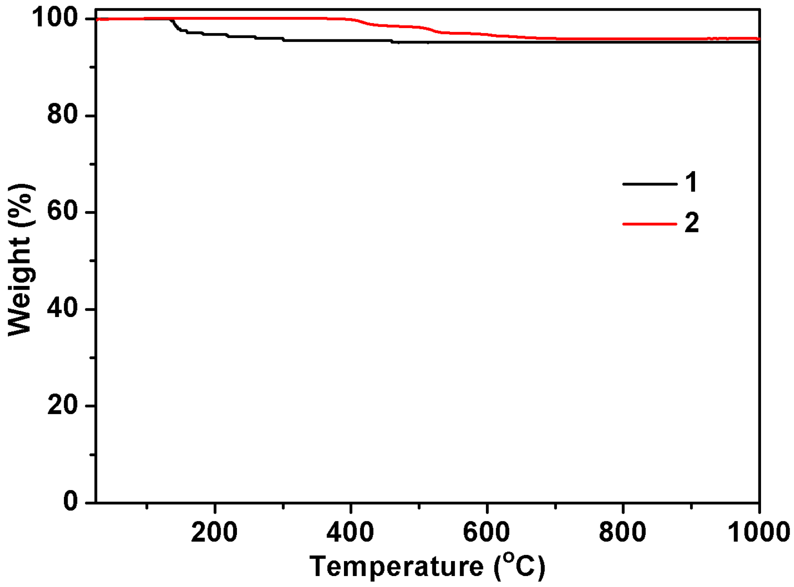

Figure S6. TG curves of **1** and **2**.
